# Research progress on the role of PKM2 in the immune response

**DOI:** 10.3389/fimmu.2022.936967

**Published:** 2022-07-28

**Authors:** Chunyan Liu, Chenchen Liu, Rong Fu

**Affiliations:** Department of Hematology, Tianjin Medical University General Hospital, Tianjin, China

**Keywords:** PKM2, immune response, metabolic reprogramming, inflammatory diseases, proinflammatory cytokines

## Abstract

Pyruvate kinase (PK) is a key enzyme that catalyzes the dephosphorylation of phosphoenolpyruvate (PEP) into pyruvate, and is responsible for the production of ATP during glycolysis. As another important isozyme of PK, pyruvate kinase M2 (PKM2) exists in cells with high levels of nucleic acid synthesis, such as normal proliferating cells (e.g., lymphocytes and intestinal epithelial cells), embryonic cells, adult stem cells, and tumor cells. With further research, PKM2, as an important regulator of cellular pathophysiological activity, has attracted increasing attention in the process of autoimmune response and inflammatory. In this re]view, we examine the contribution of PKM2 to the human immune response. Further studies on the immune mechanisms of PKM2 are expected to provide more new ideas and drug targets for immunotherapy of inflammatory and autoimmune diseases, guiding drug development and disease treatment.

## Introduction

Pyruvate kinase (PK) is a key enzyme that catalyzes the dephosphorylation of phosphoenolpyruvate (PEP) into pyruvate and is responsible for ATP production during glycolysis. In contrast to mitochondrial respiration, ATP production by PK is independent of oxygen supply and allows the organs to survive under hypoxic conditions. According to different metabolic functions of tissues, the expression levels of different isozymes of pyruvate kinase vary substantially in their kinetics and regulatory mechanisms, and these isozymes are mainly divided into PKL, PKR, PKM1, and PKM2 isoforms.

Besides existing in cells with high levels of nucleic acid synthesis, PKM2 is also expressed in some differentiated tissues, including adipose tissues, lung tissues, the retina, and islets. The enzymatic activity of PKM2 is regulated by allosteric effects and intracellular signal transduction. PKM2 functions in a variety of pathways, including aerobic glycolysis, intranuclear signal transduction, protein synthesis, and protein interactions (1). Previous studies on PKM2 in China and other countries have mainly focused on the effect on the metabolism of tumor cells (2–5). With further research, as an important regulator of cellular pathophysiological activity, the role of PKM2 in the autoimmune response and inflammatory process is attracting increasing attention (6). In this study, we reviewed the role of PKM2 in the immune response of the body.

## PKM2 participates in metabolic reprogramming

Although the role of PKM2 in tumors has been studied in depth, studies have shown that as a key regulator of immune cell metabolism and function, PKM2 plays a role in the maintenance of autoimmune homeostasis through regulation of the Warburg effect. Understanding the complex interactions between cell signal transduction pathways and metabolic pathways has become an important focus of research in the cancer field and recently in the field of inflammation and autoimmune diseases. Inflammatory reactions and autoimmune responses are energy-intensive processes. A large number of immune cells in the surrounding microcirculation are involved in activation and recruitment and in a drastic transition from quiescence to a highly active metabolic state. Therefore, metabolic reprogramming to direct nutrients to effectively produce ATP and synthesize macromolecules is necessary for proinflammatory mediator production, cytoskeletal rearrangements, and immune cell proliferation. At this stage, these highly active immune cells undergo metabolic transformation from oxidative phosphorylation to aerobic glycolysis, similar to the Warburg effect found in tumor cells. In fact, the enzymes originally involved in the regulation of cell metabolism and their regulatory agents also play a key role in regulating immune cell function. Therefore, regulation of immune cell metabolism has become a new target for inflammatory diseases and autoimmune diseases.

High PKM2 expression promotes the accumulation of upstream glycolysis metabolites and activates alternative pathways for anabolism, such as the synthesis of glycerol or entry into the pentose phosphate pathway to produce NADPH, inhibition of reactive oxygen species (ROS) production, and participation in nucleic acid synthesis. The mutual transformation of PKM2 in activated tetramers and inhibitory dimers plays a critical role in the adaptation of immune cells to changing oxygen content and nutrient conditions. In the cytoplasm, the tetrameric form of PKM2 can interact with other glycolytic enzymes (such as hexokinase, glyceraldehyde 3-P dehydrogenase, phosphoglycerol transferase, and enolase) and protein kinase cascade components (such as RAF, MEK, ERK, and RNA in the glycolytic enzyme complex). The high affinity of PKM2 in tetramer form with PEP and the close spatial conformation with other glycolytic enzymes in the glycolytic enzyme complex can promote efficient conversion of glucose into pyruvate and lactate (7).

## PKM2 participates in the innate immune response

**Figure 1:**
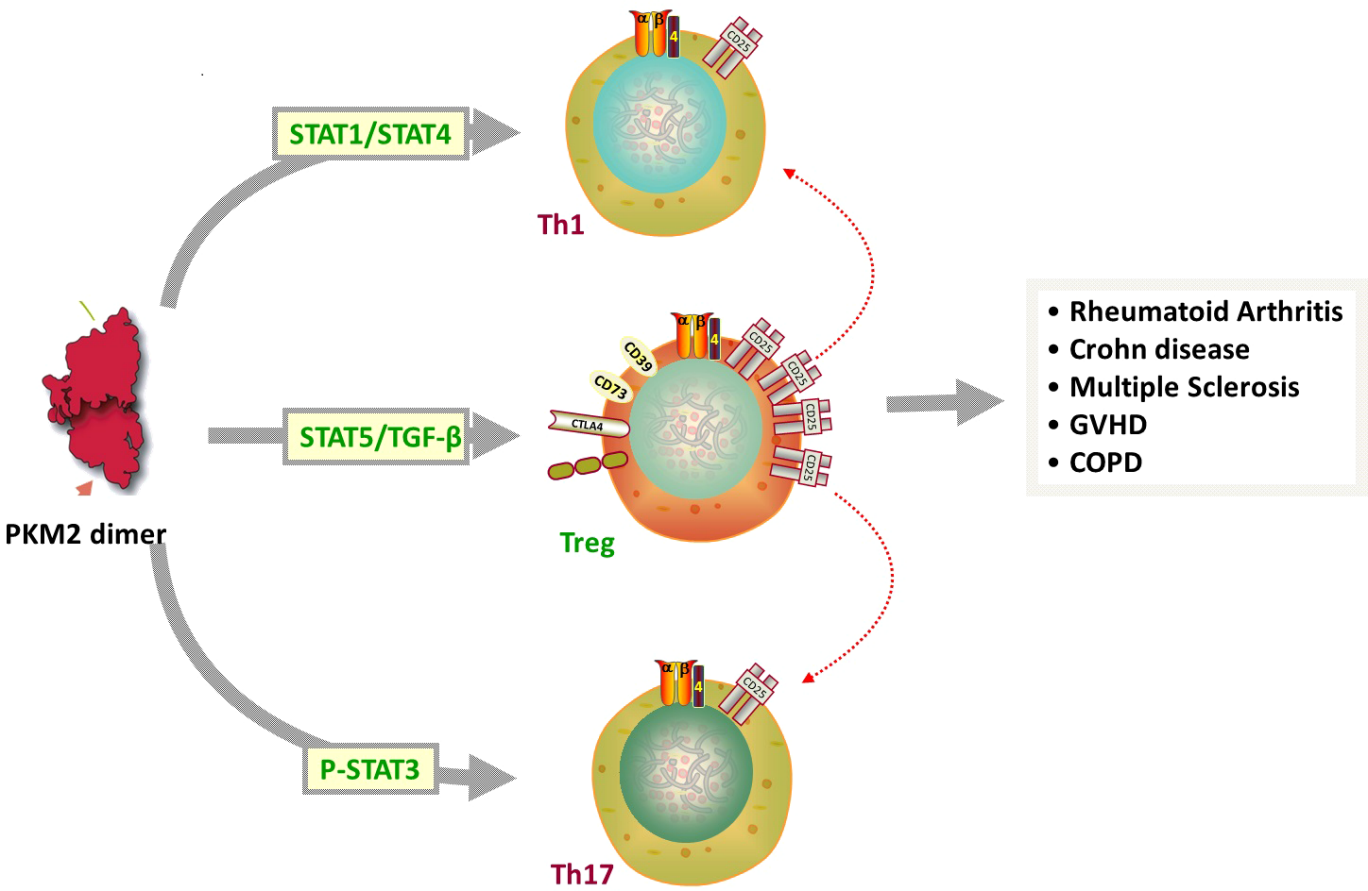
1) In its dimeric form, PKM2 can translocate to the nucleus to regulate the expression of numerous proteins involved in complex biological and biochemical processes. 2) LPS induces translocation of PKM2 into the nucleus and forms a transcription complex with HIF-1α, increasing the expression of the proinflammatory cytokines IL-1β and TNFa PKM2 can activate and interact with HIF-1α to modulate high mobility group box-1 (HMGB1) released by activated macrophages. 3) DASA-58 and TEPP-46 can convert PKM2 into a tetramer conformation, effectively inhibiting LPS-induced nuclear translocation and subsequent expression of IL-1β and a series of other HIF-1α-dependent genes. 4) Knockdown or inhibition of PKM2 expression using shikonin can significantly reduce the release of HMGB1 in activated macrophages.

PKM2 can increase the expression of the proinflammatory cytokines interleukin 1β (IL-1β) and tumor necrosis factor-α (TNFa) by participating in the innate immune response (8). Both IL-1β and TNFα are considered important effector molecules in causing autoimmune diseases and metabolic diseases. Current evidence indicates that PKM2 expression is significantly increased in lipopolysaccharide (LPS)-activated macrophages, mainly in the less active monomer/dimer conformation and phosphorylation status. At the same time, LPS induces translocation of PKM2 into the nucleus and forms a transcription complex with hypoxia-inducible factor (HIF)-1α, which then directly binds to the IL-1β promoter gene and activates its transcription, highlighting the important function of PKM2 in metabolic reprogramming and the regulation of gene expression in activated macrophages. On the other hand, DASA-58 and TEPP-46 can convert PKM2 into a tetramer conformation, effectively inhibiting LPS-induced nuclear translocation and subsequent expression of IL-1β and a series of other HIF-1α-dependent genes (8). In addition, evidence indicates that PKM2 can activate and interact with HIF-1α to modulate high mobility group box-1 (HMGB1) released by activated macrophages (9). HMGB1 is a ubiquitous nuclear protein that can be released by activated macrophages and used as an effective proinflammatory cytokine (10). Knockdown or inhibition of PKM2 expression using short hairpin RNA (shRNA) or shikonin can significantly reduce the release of HMGB1 in activated macrophages (11). In addition, stimulation of colon cancer cells using LPS led to increased production of TNF-α and IL-1β in a PKM2/STAT3-dependent manner (12, 13). LPS can induce nuclear translocation of PKM2 and bind to the STAT3 promoter to activate and enhance transcription. Recent studies have also demonstrated the key role of dimeric PKM2 in the hyperinflammatory behaviors of macrophages in patients with coronary artery disease (CAD) (14). The results indicated that nuclear translocation of dimeric PKM2 led to phosphorylation of STAT3 in LPS-stimulated CAD macrophages and promoted IL-1β and IL-6 transcription. The use of ML265 to immobilize PKM2 in a tetrameric conformation can prevent LPS-induced nuclear translocation and STAT3 phosphorylation (**Figure 1**).

Therefore, PKM2 appears to be a key regulator of the expression and secretion of proinflammatory mediators, showing the possibility of targeted therapy for inflammatory and infectious diseases using this protein. In addition, PKM2-mediated glycolysis promotes the activation of inflammasomes and the release of IL-1β, IL-18, and HMGB1 by macrophages through regulation of EIF2AK2 phosphorylation in macrophages, thus promoting the development of sepsis. In a mouse model, inhibition of the PKM2-EIF2AK2 pathway protected mice from lethal endotoxemia and microbial sepsis (15). At the same time, increased glucose uptake and glycolytic flux in monocytes and macrophages of patients with atherosclerotic CAD promote mitochondrial ROS production, which promotes dimerization and nuclear translocation of glycolytic PKM2. Nuclear PKM2 acts as a protein kinase to phosphorylate the kinase STAT3, thereby enhancing the production of IL-6 and IL-1β to trigger systemic and tissue inflammation (14).

When activated by toll-like receptor (TLR) ligands or proinflammatory cytokines, immune cells, including neutrophils, dendritic cells (DCs), and macrophages, convert their metabolic pathways from oxidative phosphorylation to aerobic glycolysis. Krawczyk et al. (16) described this phenomenon in DCs and showed that TLR stimulation is essential for the maturation of DCs. Stimulation of DCs by ligands of TLR4, TLR2, and TLR9 may lead to increases in the glycolysis rate and glucose consumption, promote the mRNA and protein expression of glucose transporter 1 (GLUT1), and increase the production of lactic acid. This metabolic conversion is achieved through PI3K–HIF 1α-PKM2 cascade regulation (17). In a mouse model of pneumonia, PKM2 is critical for activation of LPS-stimulated DCs. The JNK-P300 signaling axis mediates the acetylation of PKM2 at K433 to relocate PKM2 to the nucleus, which induces PKM2 detetramerization associated with reduced pyruvate kinase activity and simultaneously promotes glycolysis and lipid synthesis, thereby achieving metabolic reprogramming suitable for activated DCs. Further experiments confirmed that inhibition of PKM2 detetramerization significantly inhibited the expression of IL-12p35 and reduced lung inflammation in mice (18).

## PKM2 and acquired immune response

**Figure 2:**
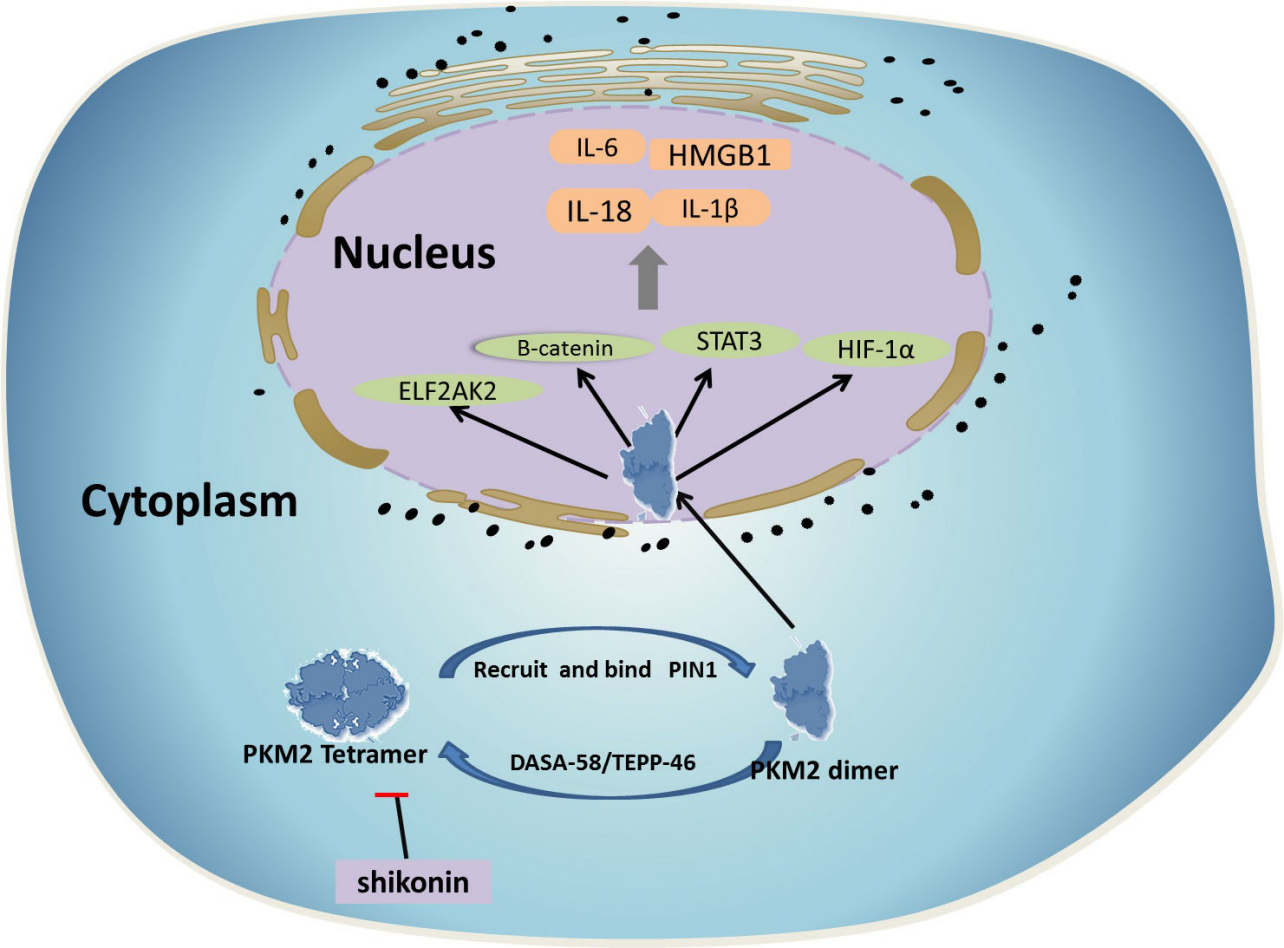
Among helper T cell subsets, PKM2 acts on the Stat family in proinflammatory T cell subset differentiation. 1) PKM2 regulates STAT1/STAT4 to induce Th1 cells formation and participates in cellular immunity. 2) PKM2 can then regulate STAT3 phosphorylation to regulate the differentiation of Th17 cells, participating in inflammation related diseases. 3) PKM2 can regulate STAT5 with TGF- β Binding, induces Tregs formation and maintains immune tolerance.

The PKM2-mediated Warburg effect not only participates in activation of innate immune cells, such as macrophage polarization (19), but also plays an important role in the acquired immune response. As an indispensable part of the acquired immune system, infection- or autoimmune-specific T cell populations are mainly derived from a common precursor—natural CD4^+^ T cells. After T cell receptor (TCR) activation, these natural CD4^+^ T cells differentiate into multiple lines of T helper (Th) cells based on extracellular factors and environmental signals, including Th17 cells and regulatory T cells (Tregs). Among helper T cell subsets, PKM2 acts on the Stat family in proinflammatory T cell subset differentiation. Pkm2 regulates STAT1/STAT4 to induce Th1 formation and participates in cellular immunity (20). Pkm2 can then regulate STAT6 and STAT3 phosphorylation to regulate the differentiation of Th2 and Th17 cells, respectively, participating in inflammation related diseases. Finally, pkm2 can regulate STAT5 with TGF-β Binding, induces Tregs formation and maintains immune tolerance (21) (**Figure 2**). Th17 cells produce IL-17, a proinflammatory cytokine. Th17 cells not only play important roles in infectious diseases but also participate in the occurrence and development of various autoimmune diseases such as rheumatoid arthritis, psoriasis, autoimmune uveitis, diabetes mellitus, and multiple sclerosis. Tregs also show anti-inflammatory properties, and insufficient numbers or functions of Tregs will lead to inflammation or progression of autoimmune diseases (22).

By observing the metabolic process of proinflammatory M1 macrophages and inflammatory T cells (such as Th17 cells), we found that the above cells all showed increased levels of PKM2 and HIF-1α transcription (23). Similarly, anti-inflammatory M2 macrophages with a high oxidative phosphorylation rate have a metabolic status similar to that of Tregs. A reduction in PKM2 expression may inhibit the glycolysis reaction, promote Treg cells differentiation, and reduce the level of Th17 cell differentiation (24). Therefore, as an important link in regulating glycolysis, PKM2 plays an important role in the immune response of the body.

In addition, evidence indicates that PKM2 may also be involved in the negative feedback regulation of inhibiting Forkhead box protein P3 (FOXP3) and Treg cells function under inflammatory conditions. In the autoimmune encephalomyelitis model, translocation of PKM2 into the nucleus indirectly regulates the metabolic reprogramming and differentiation of Th17 cells through interaction with PSTAT3 and contributes to disease progression (21). In patients with chronic inflammation, such as rheumatoid arthritis, the PKM2/STAT3 signaling pathway participates in the production of IL-17 and fatty acids in CD4^+^ T cells under a lactate accumulation environment, thereby causing retention of CD4^+^ T cells and promoting disease progression (18).

Consistent with these observations, recent studies have reported increased expression of PKM2 in many diseases where autoimmune and inflammatory factors are involved in pathogenesis (25–27). PKM2 expression in intestinal tissue is high in patients with Crohn’s disease and is positively correlated with the disease activity score or serum inflammatory markers. In addition, elevated PKM2 levels were found in stool samples from patients with active Crohn’s disease, suggesting that this protein may be a useful noninvasive marker of inflammatory bowel disease (11). In addition, proteomic analysis showed that PKM2 is one of 33 overexpressed proteins found in synovial tissues of patients with rheumatoid arthritis (28). The latest research shows that in the Freund’s adjuvant (CFA)-induced rat model of inflammatory pain, PKM2 is also involved in the glycolysis process in astrocytes and participates in the formation of chronic inflammatory pain (29). PKM2 has also been shown to have higher expression in patients with severe coronavirus disease 2019 (COVID-19), suggesting that increased PKM2 is involved in the metabolic reprogramming process of patients with severe COVID-19, thus participating in the immune response induced by COVID-19 (30). Tetramerization of PKM2 in nasal epithelial cells has also been shown to induce downstream inflammatory signals by partially activating STAT3 and inducing IL-1β production in a mouse model to participate in the pathogenesis of airway hyperresponsiveness (31). In a mouse model of renal fibrosis, PKM2 was demonstrated to be overexpressed in activated fibroblasts to further induce renal interstitial fibrosis, participate in the metabolic reprogramming of fibroblasts, increase the level of glucose metabolism, and promote the progression of renal fibrosis (32). PKM2 is also associated with the pathogenesis of pulmonary arterial hypertension (PAH). Pulmonary vessels and circulating progenitor cells isolated from patients showed down regulation of microRNA-124 (miR-124), resulting in abnormal metabolism and proliferation of PAH epithelial cells through PTPB1 and PKM1/PKM2, which affects the normal metabolism of pulmonary epithelial cells and promotes the formation of pulmonary hypertension (33).

## Interaction between PKM2 and proteins

PK has roles in many aspects of the immune response. Neutrophils lacking PK activity lose their intracellular killing effect, and affected patients are prone to frequent staphylococcal infection, indicating that PK gene deficiency will affect the body’s innate immune response and anti-infective capacity (34). The latest research shows that PKM2 is regulated by interacting with suppressor of cytokine signalling-3 (SOCS3), thereby interfering with the antigen presentation ability of DCs (35). Glucose is a continuous driving force that ensures maintenance of the normal function of immune cells. During interactions with antigen-presenting cells, T cells not only increase glucose uptake but also accelerate the rate of glycolysis. The abnormal glycolysis caused by PK deficiency can weaken the normal function of immune cells and interfere with the ability of cells to eliminate intracellular pathogens. PKM2 can also stimulate the maturation of DCs and convert their metabolic pathways from oxidative phosphorylation to aerobic glycolysis (16).

Another piece of evidence that PKM2 participates in the immune response is that PKM2 can interact with IgE receptors in cells, thereby inhibiting its activity (36). A follow-up study suggested that this interaction may lead to degranulation of mast cells involved in allergic reactions (37). Recently, mast cell degranulation has been reported to be involved in the inhibition of PKM2 activity mediated by Fcγ receptor β, thereby exhibiting mutual inhibition between PKM2 activity and mast cell degranulation (36, 38). Another study suggested that overexpression of PKM2 and annexin I proteins is conducive to the formation of granules of TNFα and other mediators in mast cells in allergic diseases. Therefore, PKM2 plays an important role in the body’s immune response to allergens (39). PKM2 also interacts with homocysteine (Hcy). Overactivation of PKM2 in B cells by Hcy induces metabolic reprogramming of B cells, enhances the glycolytic activity of B cells, promotes B cell proliferation and antibody secretion, and accelerates the development of atherosclerosis caused by hyperhomocysteinemia (40).

In addition, some pathogen proteins enhance their pathogenicity by interacting with PKM2, such as the staphylococcal protein Opa, human papillomavirus (HPV), and hepatitis C virus (HCV) (41–44). PKM1 is considered an antigenic substance in Tourette syndrome and can be a target of the autoimmune response after staphylococcal infection (45). In the BALB/c mouse model, the presentation of PKM1/M2 peptide by DCs induces allergic myositis (46). Studies on Bloom syndrome have shown that intracellularly released dimeric PKM2 can become an antigenic substance and induce a series of autoimmune responses (47). The proteins secreted by TaPin1 parasites can stabilize host PKM2 proteins, thereby affecting HIF-1α activity and thus affecting glucose metabolism and cell differentiation in the host (48).

PKM2 plays an important role in the proliferation, angiogenesis, and metabolic reprogramming induced by human cytomegalovirus (HCMV)-encoded chemokine receptor US28. PKM2 activates the HIF-1α/PKM2 feedforward loop and maintains HIF-1α protein stability in fibroblast and glioblastoma cells through activation of various proliferative and angiogenic signaling pathways (40). In addition, PKM2 can also modify the peptides produced in the cell apoptosis process. If these substances are not effectively removed, they are released from cells and are taken up, processed, and presented to T or B cells by DCs as immunogens. PKM2 also acts on some viral RNA polymerases. The C-terminal region of PKM2 proteins interacts with the C-terminus of viral protein subunits to enhance viral pathogenicity; on the other hand, PKM2 catalyzes the transfer of phosphate groups from PEP to ADP. This process produces one molecule of pyruvate and one molecule of ATP, providing raw materials and energy for virus proliferation, enhancing viral replication, and further activating DC function (49). At the same time, phosphorylation of viral protein tyrosine residues causes allosteric transformation of PKM2 and further enhances glucose metabolism. PKM2 activity can be regulated by phosphotyrosine growth signal transduction in cells. The interaction between PKM2 and phosphotyrosine-containing proteins and peptides results in the release of bound fructose-1,6-bisphosphate (FBP) from PKM2, which enhances PKM2 expression through a feedback mechanism (50, 51).

Endothelial nitric oxide synthase (eNOS) interacts with S-nitroso PKM2, thereby reducing PKM2 activity. Inhibition of PKM2 can increase substrate flux through the pentose phosphate pathway to produce reducing equivalents (NADPH and GSH) and prevent oxidative stress, which delays the development of cardiovascular disease (52). PKM2 is an essential enzyme in S-adenosylmethionine synthesis in endothelial cells (ECs). DNA methylation is insufficient in the absence of PKM2, which causes suppression of endogenous retrovirus elements (ERV) and activation of antiviral innate immune signals, leading to an inflammatory response (53). Therefore, PKM2 is closely associated with cellular immunity.

## Summary

Over the past few years, metabolism and immunology have become two different fields of investigation. However, metabolism and immunology are believed to have extensive interactions in many aspects. The concept of metabolic reprogramming as an important driving mechanism of immune responses mainly focuses on how the metabolic state of immune cells directly affects their activities and functions. In recent years, PKM2 has not only emerged as a key regulator of metabolic reprogramming but also been found to be involved in regulating the transcription of key genes in immune cells. PKM2 expression levels and enzyme activities can be regulated at multiple levels, including the transcription level and posttranslational modification level, and can change conformational stability through allosteric regulation (11, 54).

PKM2 has also been confirmed to be a target of treatments for certain inflammatory conditions. PKM2 has been shown to be involved in relieving synovial inflammation in destabilization of the medial meniscus (DMM) mouse models and air pouch models and in relieving pain gait patterns in DMM mice through low-intensity pulsed ultrasound (LIPUS). LIPUS achieves this effect by inhibiting the production of mature IL-1β *in vitro* and *in vivo*, upregulating the macrophage autophagy level, and accelerating the formation of the SQSTM1 (sequestosome 1)-PKM (pyruvate kinase, muscle) complex in macrophages treated with lipopolysaccharide (LPS)-adenosine triphosphate (ATP) (55). PKM2 participates in the occurrence and development of pulmonary arterial hypertension in extrapulmonary fibroblasts. Inhibition of PKM2 can reverse the glycolysis status of the hypertensive pulmonary arterial wall (PH-Fib), reduce its cell proliferation, and decrease the expression of IL-1β in macrophages, thus delaying the progression of pulmonary hypertension (56). *In vitro*, activation of PKM2 increases glucose metabolic flux by partially increasing glycolytic flux and PGC-1α mRNA in cultured podocytes, thereby inhibiting the production of toxic glucose metabolites, inducing mitochondrial production, and restoring mitochondrial functions to prevent diabetic nephropathy (DN) (57). Dimerization of PKM2 can promote the dopamine D2 receptor (DRD2) in astrocytes to promote dopamine biosynthesis through GSH synthesis regulated by PKM2-mediated Nrf2 transactivation, thus providing a potential target for Parkinson’s disease (58). Further studies on the immune mechanism of PKM2 are expected to provide more new ideas and targets of drug action for immunotherapy for clinical inflammatory diseases and autoimmune diseases and guide drug development and disease treatment.

## Author contributions

CYL and CCL contributed equally to this manuscript. All authors contributed to the article and approved the submitted version.

## Funding

This work was supported by the National Natural Science Foundation of China (81970115, 81800120, 81870101, 81800119, 81900125, and 81970116).

## Acknowledgments

We thank James P. Mahaffey, PhD, from Liwen Bianji (Edanz) (www.liwenbianji.cn/), for editing the English text of a draft of this manuscript.

## Conflict of interest

The authors declare that the research was conducted in the absence of any commercial or financial relationships that could be construed as a potential conflict of interest.

## Publisher’s note

All claims expressed in this article are solely those of the authors and do not necessarily represent those of their affiliated organizations, or those of the publisher, the editors and the reviewers. Any product that may be evaluated in this article, or claim that may be made by its manufacturer, is not guaranteed or endorsed by the publisher.

## References

[1] Pan T Xu J Zhu Y. Self-renewal molecular mechanisms of colorectal cancer stem cells. Int J Mol Med (2017) 39(1):9–20. doi: 10.3892/ijmm.2016.2815 27909729 PMC5179189

[2] Yang L Zheng S Liu Q. Plasma−derived exosomal pyruvate kinase isoenzyme type M2 accelerates the proliferation and motility of oesophageal squamous cell carcinoma cells. Oncol Rep (2021) 46(4):216. doi: 10.3892/or.2021.8167 34396437 PMC8377463

[3] Song L Zhang W Chang Z Pan Y Zong H Fan Q. miR-4417 targets tripartite motif-containing 35 (TRIM35) and regulates pyruvate kinase muscle 2 (PKM2) phosphorylation to promote proliferation and suppress apoptosis in hepatocellular carcinoma cells. Med Sci Monit (2017) 23:1741–50. doi: 10.12659/MSM.900296 PMC5398329 28394882

[4] Wang X Fa XE. Knockdown of UCA1 inhibits viability and glycolysis by suppressing PKM2 expression through the mTOR pathway in non-small cell lung cancer cells. RSC Adv (2018) 8(19):10610–9. doi: 10.1039/C8RA00860D PMC9078902 35540445

[5] Cheng Z Yu S Qun W. Application of M2-type pyruvate kinase in cancer diagnosis and therapy. Chin J Cancer Pre Treat (2013) 20(13):1043–6.

[6] Mazurek S. Pyruvate kinase type M2: a key regulator of the metabolic budget system in tumor cells. Int J Biochem Cell Biol (2011) 43(7):969–80. doi: 10.1016/j.biocel.2010.02.005 20156581

[7] Spoden GA Mazurek S Morandell D Bacher N Ausserlechner M. J Jansen- Dürr P. Isotype-specific inhibitors of the glycolytic key regulator pyruvate kinase subtype M2 moderately decelerate tumor cell proliferation. Int J Cancer (2008) 123(2):312–21. doi: 10.1002/ijc.23512 18425820

[8] Palsson-Mcdermott E Curtis A Goel G Lauterbach MA Sheedy FJ Gleeson LE. Pyruvate kinase M2 regulates hif-1α activity and IL-1β induction and is a critical determinant of the warburg effect in LPS-activated macrophages. Cell Metab (2015) 21(2):347. doi: 10.1016/j.cmet.2014.12.005 29510100

[9] Yang L Xie M Yang M Yu Y Zhu S Hou W. PKM2 regulates the warburg effect and promotes HMGB1 release in sepsis. Nat Commun (2014) 5:4436. doi: 10.1038/ncomms5436 25019241 PMC4104986

[10] Andersson U Wang H Palmblad K Aveberger AC Bloom O Erlandsson- Harris H. High mobility group 1 protein (HMG-1) stimulates proinflammatory cytokine synthesis in human monocytes. J Exp Med (2000) 192(4):565–70. doi: 10.1084/jem.192.4.565 PMC2193240 10952726

[11] Alves-Filho JC Pålsson-Mcdermott EM. Pyruvate kinase M2: A potential target for regulating inflammation. Front Immunol (2016) 7:145. doi: 10.3389/fimmu.2016.00145 27148264 PMC4838608

[12] Xu D Liang J Lin J Yu C. PKM2: A potential regulator of rheumatoid arthritis *via* glycolytic and non-glycolytic pathways. Front Immunol (2019) 10:2919. doi: 10.3389/fimmu.2019.02919 31921178 PMC6930793

[13] Van De Wetering C Manuel AM Sharafi M Aboushousha R Qian X Erickson C. Glutathione-s-transferase p promotes glycolysis in asthma in association with oxidation of pyruvate kinase M2. Redox Biol (2021) 47:102160. doi: 10.1016/j.redox.2021.102160 34624602 PMC8502950

[14] Shirai T Nazarewicz RR Wallis BB Yanes RE Watanabe R Hilhorst M. The glycolytic enzyme PKM2 bridges metabolic and inflammatory dysfunction in coronary artery disease. J Exp Med (2016) 213(3):337–54. doi: 10.1084/jem.20150900 PMC4813677 26926996

[15] Xu F Guo M Huang W Feng L Zhu J Luo K. Annexin A5 regulates hepatic macrophage polarization *via* directly targeting PKM2 and ameliorates NASH. Redox Biol (2020) 36:101634. doi: 10.1016/j.redox.2020.101634 32863213 PMC7369618

[16] Krawczyk CM Holowka T Sun J Blagih J Amiel E DeBerardinis RJ. Toll-like receptor-induced changes in glycolytic metabolism regulate dendritic cell activation. Blood (2010) 115(23):4742–9. doi: 10.1182/blood-2009-10-249540 PMC2890190 20351312

[17] Talesa VN Ferri I Bellezza G Love HD Sidoni A Antognelli C. Glyoxalase 2 is involved in human prostate cancer progression as part of a mechanism driven by PTEN/PI3K/AKT/mTOR signaling with involvement of PKM2 and ERα. Prostate (2017) 77(2):196–210. doi: 10.1002/pros.23261 27696457

[18] Pucino V Certo M Bulusu V Cucchi D Goldmann K Pontarini E. Lactate buildup at the site of chronic inflammation promotes disease by inducing CD4(+) T cell metabolic rewiring. Cell Metab (2019) 30(6):1055–1074.e8. doi: 10.1016/j.cmet.2019.10.004 31708446 PMC6899510

[19] Palsson-Mcdermott EM O'neill LA. The warburg effect then and now: from cancer to inflammatory diseases. Bioessays (2013) 35(11):965–73. doi: 10.1002/bies.201300084 24115022

[20] Wang L Deng Z Sun Y Zhao Y Li Y Yang M. The study on the regulation of Th cells by mesenchymal stem cells through the JAK-STAT signaling pathway to protect naturally aged sepsis model rats. Front Immunol (2022) 13:820685. doi: 10.3389/fimmu.2022.820685 35197984 PMC8858840

[21] Damasceno LEA Prado DS Veras FP Fonseca MM Toller-Kawahisa JE Rosa MH. PKM2 promotes Th17 cell differentiation and autoimmune inflammation by fine-tuning STAT3 activation. J Exp Med (2020) 217(10). doi: 10.1084/jem.20190613 PMC7537396 32697823

[22] Yan L Fu R Liu H Wang H Liu C Wang T. Abnormal quantity and function of regulatory T cells in peripheral blood of patients with severe aplastic anemia. Cell Immunol (2015) 296(2):95–105. doi: 10.1016/j.cellimm.2015.04.001 25906694

[23] Corcoran SE O'neill LA. HIF1α and metabolic reprogramming in inflammation. J Clin Invest (2016) 126(10):3699–707. doi: 10.1172/JCI84431 PMC5096812 27571407

[24] Shi LZ Wang R Huang G Vogel P Neale G Green DR. HIF1alpha-dependent glycolytic pathway orchestrates a metabolic checkpoint for the differentiation of TH17 and treg cells. J Exp Med (2011) 208(7):1367–76. doi: 10.1084/jem.20110278 PMC3135370 21708926

[25] Tang Q Ji Q Xia W Li L Bai J Ni R. Pyruvate kinase M2 regulates apoptosis of intestinal epithelial cells in crohn's disease. Dig Dis Sci (2015) 60(2):393–404. doi: 10.1007/s10620-014-3189-0 24817408

[26] Chung-Faye G Hayee B Maestranzi S Donaldson N Forgacs I Sherwood R. Fecal M2-pyruvate kinase (M2-PK): a novel marker of intestinal inflammation. Inflammation Bowel Dis (2007) 13(11):1374–8. doi: 10.1002/ibd.20214 17577247

[27] Day AS Judd T Lemberg DA Leach ST. Fecal M2-PK in children with crohn's disease: a preliminary report. Dig Dis Sci (2012) 57(8):2166–70. doi: 10.1007/s10620-012-2215-3 22736014

[28] Li XJ Xu M Zhao XQ Zhao JN Chen FF Yu W. Proteomic analysis of synovial fibroblast-like synoviocytes from rheumatoid arthritis. Clin Exp Rheumatol (2013) 31(4):552–8. 23739258

[29] Wei X Jin XH Meng XW Hua J Ji FH LaNng W. Platelet-rich plasma improves chronic inflammatory pain by inhibiting PKM2-mediated aerobic glycolysis in astrocytes[J]. Ann Transl Med (2020) 8(21):1456. doi: 10.21037/atm-20-6502 33313201 PMC7723564

[30] Mcelvaney OJ Mcevoy NL Mcelvaney OF Carroll TP Murphy MP Dunlea DM. Characterization of the inflammatory response to severe COVID-19 illness. Am J Respir Crit Care Med (2020) 202(6):812–21. doi: 10.1164/rccm.202005-1583OC PMC7491404 32584597

[31] Van De Wetering C Aboushousha R Manuel AM Chia SB Erickson C MacPherson MB. Pyruvate kinase M2 promotes expression of proinflammatory mediators in house dust mite-induced allergic airways disease. J Immunol (2020) 204(4):763–74. doi: 10.4049/jimmunol.1901086 PMC6994838 31924651

[32] Yin XN Wang J Cui LF Fan WX. Enhanced glycolysis in the process of renal fibrosis aggravated the development of chronic kidney disease. Eur Rev Med Pharmacol Sci (2018) 22(13):4243–51. doi: 10.26355/eurrev_201807_15419 30024614

[33] Caruso P Dunmore BJ Schlosser K Schoors S Dos Santos C Perez-Iratxeta C. Identification of MicroRNA-124 as a major regulator of enhanced endothelial cell glycolysis in pulmonary arterial hypertension *via* PTBP1 (Polypyrimidine tract binding protein) and pyruvate kinase M2. Circulation (2017) 136(25):2451–67. doi: 10.1161/CIRCULATIONAHA.117.028034 PMC5736425 28971999

[34] Burge PS Johnson WS Hayward AR. Neutrophil pyruvate kinase deficiency with recurrent staphylococcal infections: first reported case. Br Med J (1976) 1(6012):742–5. doi: 10.1136/bmj.1.6012.742 PMC1639201 4193

[35] Zhang Z Liu Q Che Y Yuan X Dai L Zeng B. Antigen presentation by dendritic cells in tumors is disrupted by altered metabolism that involves pyruvate kinase M2 and its interaction with SOCS3. Cancer Res (2010) 70(1):89–98. doi: 10.1158/0008-5472.CAN-09-2970 19996282

[36] Oak MH Cheong H Kim KM. Activation of fc epsilon RI inhibits the pyruvate kinase through direct interaction with the gamma-chain. Int Arch Allergy Immunol (1999) 119(2):95–100. doi: 10.1159/000024183 10394100

[37] Shannahan JH Kodavanti UP Brown JM. Manufactured and airborne nanoparticle cardiopulmonary interactions: a review of mechanisms and the possible contribution of mast cells. Inhal Toxicol (2012) 24(5):320–39. doi: 10.3109/08958378.2012.668229 PMC3768266 22486349

[38] Ryu H Walker JK Kim S Koo N Barak LS Noguchi T. Regulation of M2-type pyruvate kinase mediated by the high-affinity IgE receptors is required for mast cell degranulation. Br J Pharmacol (2008) 154(5):1035–46. doi: 10.1038/bjp.2008.148 PMC2451040 18587448

[39] Kim JY Kim DY Ro JY. Granule formation in NGF-cultured mast cells is associated with expressions of pyruvate kinase type M2 and annexin I proteins. Int Arch Allergy Immunol (2008) 146(4):287–97. doi: 10.1159/000121463 18362474

[40] De Wit RH Mujić-Delić A Van Senten JR Fraile-Ramos A Siderius M Smit MJ. Human cytomegalovirus encoded chemokine receptor US28 activates the HIF-1α/PKM2 axis in glioblastoma cells. Oncotarget (2016) 7(42):67966–85. doi: 10.18632/oncotarget.11817 PMC5356532 27602585

[41] Williams JM Chen GC Zhu L Rest RF. Using the yeast two-hybrid system to identify human epithelial cell proteins that bind gonococcal opa proteins: intracellular gonococci bind pyruvate kinase *via* their opa proteins and require host pyruvate for growth. Mol Microbiol (1998) 27(1):171–86. doi: 10.1046/j.1365-2958.1998.00670.x 9466265

[42] Mazurek S Zwerschke W Jansen-Dürr P Eigenbrod E. Effects of the human papilloma virus HPV-16 E7 oncoprotein on glycolysis and glutaminolysis: role of pyruvate kinase type M2 and the glycolytic-enzyme complex. Biochem J (2001) 356(Pt 1):247–56. doi: 10.1042/bj3560247 PMC1221834 11336658

[43] Zwerschke W Mazurek S Massimi P Banks L Eigenbrodt E Jansen-Dürr P. Modulation of type M2 pyruvate kinase activity by the human papillomavirus type 16 E7 oncoprotein. Proc Natl Acad Sci U.S.A. (1999) 96(4):1291–6. doi: 10.1073/pnas.96.4.1291 PMC15456 9990017

[44] Wu X Zhou Y Zhang K Liu Q Guo D. Isoform-specific interaction of pyruvate kinase with hepatitis c virus NS5B. FEBS Lett (2008) 582(15):2155–60. doi: 10.1016/j.febslet.2008.05.033 18519040

[45] Kansy JW Katsovich L Mciver KS Pick J Zabriskie JB Lombroso PJ. Identification of pyruvate kinase as an antigen associated with tourette syndrome. J Neuroimmunol (2006) 181(1-2):165–76. doi: 10.1016/j.jneuroim.2006.08.007 PMC1853370 17011640

[46] Kawachi I Tanaka K Tanaka M Tsuji S. Dendritic cells presenting pyruvate kinase M1/M2 isozyme peptide can induce experimental allergic myositis in BALB/c mice. J Neuroimmunol (2001) 117(1-2):108–15. doi: 10.1016/S0165-5728(01)00327-7 11431010

[47] Ewald N Schaller M Bayer M Akinci A Bretzel RG Kloer HU. Fecal pyruvate kinase-M2 (tumor M2-PK) measurement: a new screening concept for colorectal cancer. Anticancer Res (2007) 27(4a):1949–52. 17649802

[48] Marsolier J Perichon M Weitzman JB Medjkane S. Secreted parasite Pin1 isomerase stabilizes host PKM2 to reprogram host cell metabolism. Commun Biol (2019) 2:152. doi: 10.1038/s42003-019-0386-6 31044177 PMC6491484

[49] Miyake Y Ishii K Honda A. Influenza virus infection induces host pyruvate kinase m which interacts with viral RNA-dependent RNA polymerase. Front Microbiol (2017) 8:162. doi: 10.3389/fmicb.2017.00162 28232820 PMC5298958

[50] Varghese B Swaminathan G Plotnikov A Tzimas C Yang N Rui H. Prolactin inhibits activity of pyruvate kinase M2 to stimulate cell proliferation. Mol Endocrinol (2010) 24(12):2356–65. doi: 10.1210/me.2010-0219 PMC2999476 20962042

[51] Christofk HR Vander Heiden MG Wu N Asara JM Cantley LC. Pyruvate kinase M2 is a phosphotyrosine-binding protein. Nature (2008) 452(7184):181–6. doi: 10.1038/nature06667 18337815

[52] Siragusa M Thöle J Bibli SI Luck B Loot AE de Silva K. Nitric oxide maintains endothelial redox homeostasis through PKM2 inhibition. EMBO J (2019) 38(17):e100938. doi: 10.15252/embj.2018100938 31328803 PMC6717893

[53] Stone OA El-Brolosy M Wilhelm K Liu X Romão AM Grillo E. Loss of pyruvate kinase M2 limits growth and triggers innate immune signaling in endothelial cells. Nat Commun (2018) 9(1):4077. doi: 10.1038/s41467-018-06406-8 30301887 PMC6177464

[54] Uguccioni M Teixeira MM Locati M Mantovan A. Editorial: Regulation of inflammation, its resolution and therapeutic targeting. Front Immunol (2017) 8:415. doi: 10.3389/fimmu.2017.00415 28458666 PMC5394768

[55] Zhang B Chen H Ouyang J Xie Y Chen L Tan Q. SQSTM1-dependent autophagic degradation of PKM2 inhibits the production of mature IL1B/IL-1β and contributes to LIPUS-mediated anti-inflammatory effect. Autophagy (2020) 16(7):1262–78. doi: 10.1080/15548627.2019.1664705 PMC7469634 31500508

[56] Zhang H Wang D Li M Plecitá-Hlavatá L D'Alessandro A Tauber J. Metabolic and proliferative state of vascular adventitial fibroblasts in pulmonary hypertension is regulated through a MicroRNA-124/PTBP1 (Polypyrimidine tract binding protein 1)/Pyruvate kinase muscle axis. Circulation (2017) 136(25):2468–85. doi: 10.1161/CIRCULATIONAHA.117.028069 PMC5973494 28972001

[57] Qi W Keenan HA Li Q Ishikado A Kannt A Sadowski T. Pyruvate kinase M2 activation may protect against the progression of diabetic glomerular pathology and mitochondrial dysfunction. Nat Med (2017) 23(6):753–62. doi: 10.1038/nm.4328 PMC5575773 28436957

[58] Wei Y Lu M Mei M Wang H Han Z Chen M. Pyridoxine induces glutathione synthesis *via* PKM2-mediated Nrf2 transactivation and confers neuroprotection. Nat Commun (2020) 11(1):941. doi: 10.1038/s41467-020-14788-x 32071304 PMC7029000

